# Brain plasticity and cognitive functions after ethanol consumption in C57BL/6J mice

**DOI:** 10.1038/tp.2015.183

**Published:** 2015-12-15

**Authors:** E Stragier, V Martin, E Davenas, C Poilbout, R Mongeau, R Corradetti, L Lanfumey

**Affiliations:** 1 Centre de Psychiatrie et Neurosciences, INSERM UMR 894, Paris, France; 2 Université Paris Descartes, UMR S894, Paris, France; 3 Pharmacologie de la circulation cérébrale EA 4475, Faculté de pharmacie Université Paris Descartes, Paris, France; 4 Department of Neuroscience, Psychology, Drug Research and Child Health, University of Florence, Florence, Italy

## Abstract

Acute or chronic administrations of high doses of ethanol in mice are known to produce severe cognitive deficits linked to hippocampal damage. However, we recently reported that chronic and moderate ethanol intake in C57BL/6J mice induced chromatin remodeling within the *Bdnf* promoters, leading to both enhanced brain-derived neurotrophic factor (BDNF) expression and hippocampal neurogenesis under free-choice protocol. We performed here a series of cellular and behavioral studies to analyze the consequences of these modifications. We showed that a 3-week chronic free-choice ethanol consumption in C57BL/6J mice led to a decrease in DNA methylation of the *Bdnf* gene within the CA1 and CA3 subfields of the hippocampus, and upregulated hippocampal BDNF signaling pathways mediated by ERK, AKT and CREB. However, this activation did not affect long-term potentiation in the CA1. Conversely, ethanol intake impaired learning and memory capacities analyzed in the contextual fear conditioning test and the novel object recognition task. In addition, ethanol increased behavioral perseveration in the Barnes maze test but did not alter the mouse overall spatial capacities. These data suggested that in conditions of chronic and moderate ethanol intake, the chromatin remodeling leading to BDNF signaling upregulation is probably an adaptive process, engaged via epigenetic regulations, to counteract the cognitive deficits induced by ethanol.

## Introduction

Drugs of abuse have been shown to alter synaptic plasticity and related neuronal function, and this damage is particularly important in case of alcohol.^1^ However, we recently reported, using adult C57BL/6J mice, that chronic and moderate ethanol consumption induced chromatin remodeling at histone level within the various promoters of the brain-derived neurotrophic factor (BDNF) through post-translational histone modifications.^2^ These epigenetic modifications were probably responsible, at least in part, for the increase in BDNF protein expression observed after such consumption and were associated to hippocampal neurogenesis stimulation controlled by BDNF TrkB receptor.^2^ In addition, it has been shown that DNA methylation that consists in the addition of a methyl group on cytosine at CpG sites leading to transcriptional gene repression^3^ was also associated with alcohol dependence.^4^ Conversely, studies have also demonstrated that DNA methylation and DNA methyltransferase enzymes were strongly impacted by ethanol in alcoholic patients.^5, 6^

Interestingly, epigenetic regulations of gene transcription, in particular that of *Bdnf*, have been shown to have a key role in cognitive performances: a decrease in *Bdnf* methylation has been linked to memory enhancement in rats, suggesting that epigenetic modifications within this gene could be a crucial mechanism involved in memory consolidation.^7^ However, ethanol could impact BDNF-related cognition not only through epigenetic modulations but also by altering its transduction regulation. BDNF TrkB receptor cellular pathways involve the activation of several kinases. Among them, CREB phosphorylation signaling pathways are known to be inhibited by ethanol exposure,^8^ and an increase in AKT activation without changes in ERK activation has been reported in the hippocampus of ethanol-avoiding versus ethanol-preferring rats.^9^ Considering the implication of these signaling pathways controlled—at least in part—by BDNF in the regulation of cognition^10^ and in anxiety-like behaviors,^11^ they could similarly exert a central role in ethanol-induced behavioral, structural and functional adaptations.

In order to further characterize the epigenetic modification induced by chronic free-choice ethanol consumption in C57BL/6J mice and the functional consequences of these ethanol-induced epigenetic regulations, we performed a series of investigations aimed at analyzing DNA methylation within the *Bdnf* gene and BDNF intracellular pathways that involve the activation of TrkB receptor-dependent kinases. We showed that ethanol consumption decreased DNA methylation within the *Bdnf* gene in CA1 and CA3 subfields of the hippocampus, upregulated TrkB-dependent BDNF intracellular signaling pathways, but led to some alterations in learning, memory and perseveration behavior. These data suggested that ethanol-induced neuroplasticity involving BDNF signaling pathway could be an adaptive response to the cognitive impairments induced by ethanol intake.

## Materials and Methods

### Animals and the free-choice ethanol paradigm

Adult male (8-week old) C57BL/6J mice (Charles River Laboratories, l'Arbresle, France) were housed under standard laboratory conditions (22±2 °C, 60% relative humidity, 12–12 h light–dark cycle (0700–1900 hours), and food and water *ad libitum*) for at least 1 week before starting a free-choice ethanol paradigm (‘ethanol' mice) or to water only (‘water' mice), as described in Supplementary Materials and Methods.^2, 12, 13^ Separate groups of mice were subjected to the free-choice ethanol paradigm or water and used for each series of experiments (except where indicated). The number of mice used and the quantity of ethanol consumed for each set of experiments are indicated in Supplementary Table S1.

All experiments were performed in strict conformity with the institutional guidelines in compliance with the French and European Communities Directives for use of animals in biomedical research (Service de Protection et Santé Animales, Préfecture de Police, Authorization #B-75-977, Directive 2010/63/UE, 22/09/2010 protocol authorization #00966.02).

### TrkB antagonist treatment

Mice (*n*=30) from ‘water' and ‘ethanol' groups were randomly distributed to receive either a TrkB receptor antagonist ANA-12 (Maybridge, Thermo Fisher Scientific, Illkirch, France) or its vehicle (1% dimethyl sulfoxide, 1% Tween 80 dissolved in 0.9% NaCl solution). Treatment lasted for the whole stabilization period (10% ethanol in drinking water), and consisted of daily (1800 hours) intraperitoneal injections of either vehicle or ANA-12 (0.5 mg per kg body weight).^14^ Volumes of ethanol and water intakes were measured every other day.

### Reverse transcription and quantitative PCR

At the end of the free-choice ethanol or water exposure (day 21), animals were killed between 1000 and 1200 hours by cervical dislocation and hippocampi were quickly removed, frozen in liquid nitrogen and stored at −80 °C until real-time reverse transcription and quantitative PCR determinations as described in Supplementary Materials and Methods. Primers used are listed in Supplementary Table S2. Gene expression was normalized by reference to the housekeeping genes *hypoxanthine phosphoribosyltransferase* (*hprt*) and *β-actin*. The 2^ΔΔCT^ (delta-delta comparative threshold) method was used to quantify the fold change in ‘ethanol' compared with ‘water' mice.

### Methylated DNA immunoprecipitation

At the end of the free-choice ethanol or water exposure (day 21), animals were killed between 1000 and 1200 hours by cervical dislocation; brains were removed, frozen in isopentane at −35 °C and processed to laser-assisted microdissection as described in Supplementary Materials and Methods. Genomic DNA from microdissected tissues of the hippocampus was extracted following the manufacturer's instructions (NucleoSpin Tissue kit, Macherey Nagel, Hoerdt, France) and DNA concentration was quantified using Nanodrop (Fisher Scientific, Illkirch, France). Methylated DNA immunoprecipitation was performed on 2 μg of genomic DNA as described in Supplementary Materials and Methods. A volume of 1 μl of immunoprecipitated DNA and input was used for quantitative PCR analysis with *Bdnf* CpG island primers listed in Supplementary Table S3 with the following program using KAPA SYBR FAST qPCR Kit (Kapa Biosystems, CliniSciences, Nanterre, France): 95 °C, 3 min; followed by 40 cycles of 95 °C, 15 s; 60 °C, 30 s; and 72 °C, 30 s. The ratio immunoprecipitated DNA/input was then calculated to define the enrichment of methylated DNA for each CpG island.

### Immunoblotting

Mice were killed as described above and bilateral hippocampi were rapidly dissected out and then stored at −80 °C until use for western blotting as detailed in Supplementary Materials and Methods. Antibodies used were anti-ERK1/2 (1/5000; 05-1152, Millipore, Molsheim, France), anti-P-ERK1/2 at threonine 183 and tyrosine 185 (1/500; M9692, Sigma-Aldrich, Saint Quentin Fallavier, France), anti-AKT1/2/3 (1/1000; 9272S, Cell Signaling, Ozyme, Saint Quentin Yvelines, France), anti-P-AKT1/2/3 at serine 473 (1/1000; 4051S, Cell Signaling, Ozyme), anti-CREB (1/1000; 4820S, Cell Signaling, Ozyme), anti-P-CREB at serine 133 (1/1000; 9198S, Cell Signaling, Ozyme) or β-actin (1/500; SC-81178, Santa Cruz, Heidelberg, Germany). The relevant immunoreactive bands were quantified using ImageJ software (National Institutes of Health, Bethesda, MD, USA) and normalized with the total protein expression. Data normalized for each blot were expressed as percentage of ‘water' mice.

### Long-term potentiation experiments

#### Preparation of hippocampal slices and electrophysiological recordings

Hippocampal slices were obtained as previously described.^15^ Field excitatory postsynaptic potentials (fEPSPs) were evoked by electrical stimulation of the Schaffer collateral/commissural pathway and extracellularly recorded from the stratum radiatum of the CA1 region. Synaptic strength was measured as the slope of the initial falling phase of the fEPSP, following the afferent volley, as described in Supplementary Materials and Methods.

#### LTP protocol

Long-term potentiation (LTP) of synaptic responses was induced by theta-burst stimulation consisting of a single train of either 5 or 10 bursts of 5 stimuli (100 Hz intraburst frequency, 5 Hz burst frequency), called TB5 and TB10, respectively, and the magnitude of LTP was quantified as the fractional increase in fEPSP slope recorded 1 h after by TB5 stimulation and 15 min after a subsequent TB10 stimulation of the afferent pathway, as described in Mlinar *et al.*^16^ and as detailed in Supplementary Materials and Methods.

### Behavioral testing

The behavioral tests were conducted at the end of the free-choice ethanol paradigm. Animals had access *ad libitum* to ethanol and water for ‘ethanol' mice and water only for ‘water' mice during all the duration of the tests.

#### Fear conditioning test

The fear conditioning paradigm, conducted between 0900 and 1200 hours, consisted of 3 consecutive days of behavioral testing and was conducted in a chamber (26 × 18 × 22 cm), housed in a sound-attenuating box, with aluminum sidewalls and Plexiglas rear and front walls, and a stainless steel grid floor (MED Associates, St. Albans, VT, USA). On the first day, the mouse was placed into the dark conditioning chamber with vanilla odor (1%) and allowed to acclimate for 3 min. Then, a tone (conditioned stimulus (CS), 2.5 kHz, 85dB) was presented for 30 s and immediately after, a shock (unconditioned stimulus (US)) of 0.75 mA for 2 s was given, followed by an intertrial interval (ITI) of 2 min. The sessions CS–US–ITI were repeated six times. For the contextual fear test, mice were returned to the same conditioning chamber odorized with vanilla, 24 h after the training phase, and allowed to explore for 20 min without presentation of the CS or the US. The third day, the mice were tested for cued fear memory by placing them in the chambers with modified context (no vanilla odor, modification of designs to the cage walls as well as light into the chamber). The CS alone was presented 10 times, separated by 2-min ITIs. All sessions were recorded using infrared cameras and controlled by a computerized system interface (MED Associates). Freezing behavior, defined as complete absence of movements except for respiration, was measured manually every 2 s and expressed as percentage of time spent freezing.

#### Novel object recognition tests

The novel object recognition task chamber was a black PVC arena (30 × 30 × 30 cm) and the objects used were made in easy-to-clean plastic materials, having a size of about 4 × 10 × 4 cm, selected on the basis of preference tests. Room lighting was kept at 15 lux. Mice were given a 2-day habituation session in the testing chamber for 15 min (between 0900 and 1200 hours). Twenty-four hours later, during the first phase (acquisition phase), two identical objects (A and A') set in the right and left corners of the box, at a distance of 7 cm from the walls. The mouse was placed in the testing chamber and was allowed to freely explore the objects for 5 min. The mouse was then replaced in its home cage. After 1 h, the mouse returned in the testing chamber where one of the two objects (A') was replaced by a novel one (B). The mouse was allowed to freely explore for 5 min (recognition phase 1). The same procedure was replicated after a 24-h interval. The mouse was back to the testing chamber with two objects: one was the same as that during the acquisition phase (A) and a novel (C) object replaced A' (recognition phase 24). The mouse was allowed to explore the chamber for 5 min. For each mouse, the time spent interacting with each object during the recognition phases at 1 h and 24 h was video recorded and analyzed by an experimenter unaware of the group assignment. The corrected mnesic index was calculated as follows: (time spent exploring the novel object−time spent exploring the well-known object)/total time spent exploring objects. Animals able to discriminate between the well-known and new object should have a mnesic index above 0.

#### Barnes maze test

This test was conducted between 0900 and 1500 hours. The apparatus consisted of a white circular platform, 80 cm in diameter that was elevated 50 cm above the floor. Along its perimeter were 18 evenly spaced holes, each 5 cm in diameter and located 2 cm from the platform edge. The maze had one removable escape box (8 × 5 × 5 cm) that could be fitted under any of these holes. The maze was located in a white-walled room and visual cues of distinct colors and forms were situated around the maze, off the platform and were kept constant throughout the experiment. All sessions were performed on a wet platform under a room lightning of 400 lux to increase the mouse aversion for the platform. Sessions were recorded using a video-tracking system (Viewpoint, Lyon, France) and the platform was virtually divided into six quadrants, each containing three holes. Both the platform and the escape box were wiped thoroughly between each mouse session and the surface of the platform was moistened again to avoid mice using olfactory cues to solve the task.

The day before the learning sessions, mice were habituated to the apparatus. They were first placed into the escape box for 1 min and were then left free to explore the platform for 3 min and guided to the escape box. Learning sessions were initiated by placing a mouse at the middle of the maze and allowed exploring the maze for a maximum of 3 min. If the mouse failed to find the escape box during that period, it was gently guided into the escape hole where it was kept for at least 30 s. The escape box remained in its original position for subsequent sessions. The mice were trained for 9 days with 2 sessions per day, and the number of errors, the latency and the distance traveled before finding the escape box were measured.

The mice were then tested after the last training for spatial memory in a context in which they necessarily had only spatial cues for navigation. In this session the escape box was removed, the mouse was allowed exploring the maze for 30 and the time spent in the quadrant that previously contained the escape box was quantified.

The reversal learning was then performed during 4 days with 3 sessions per day. The location of the escape box was moved 120° from its original position. The mouse was placed in the middle of the platform and was allowed exploring during 3 min. Likewise, the number of errors, the latency and the distance traveled before finding the escape box were measured.

The reversal probe test was conducted immediately after the last reversal learning session. The escape box was removed and the mouse was allowed to explore the maze for 30 s. The time spent in the first quadrant containing the original position of the escape box was quantified. The reversal probe test allowed the quantification of perseveration at the original hole and reflected the cognitive flexibility needed to locate the escape box in its new location. As the platform was digitally divided into six quadrants, the random percentage of time spent in each quadrant should be equal to 16.67% in untrained normal mice. Therefore, the percent of time spent in the first target quadrant, which originally contained the escape hole, was compared with the theoretical value of 16.67% using one-sample *t*-test.

In each step of Barnes maze, mice that did not perform the task (no exploration, immobility on the platform during more than 30 s) were excluded for the rest of the experiment.

#### Anxiety-related behaviors and locomotor activity

Investigations for analyzing the effects of chronic ethanol on anxiety-related behaviors, on locomotor activity and on motor coordination were performed using classical tests (elevated plus maze, open field and rotarod). These tests, conducted between 0900 and 1200 hours, are detailed in Supplementary Materials and Methods.

### Statistical analyses

Unless otherwise indicated, values in the text and figure legends are means±s.e.m. Data from behavioral testing were analyzed using Student's *t*-test, except data from the novel object recognition test, the conditioning phase of the fear conditioning test, the learning sessions and the reversal learning of Barnes maze test, which were analyzed by a two-way analysis of variance (ANOVA; ethanol × time) with repeated measures followed by a Bonferroni's multiple comparison test when appropriated. Reversal probe test from Barnes maze test was analyzed using a one-sample *t*-test and data were compared with the theoretical value of 16.67. Data from LTP experiments were analyzed using Mann–Whitney or Wilcoxon tests, as appropriate. Methylated DNA immunoprecipitation assays were analyzed with a Kruskal–Wallis test followed by a Dunn's multiple comparison test. Reverse transcription and quantitative PCR data were analyzed using a one-way ANOVA followed by a Bonferroni test for multiple comparisons. Immunoblotting quantifications were analyzed using an unpaired *t*-test. Prism 4 software (GraphPad, San Diego, CA, USA) was used for all statistical analyses. Statistical significance was set at *P*⩽0.05.

## Results

### Ethanol intake

Mice were offered ethanol (at increasing concentration, from 3 to 10% v/v) versus water for 21 days. Total fluid intake in the ‘ethanol' group (145.40±13.48 g kg^−1^ per day, *n*=86) did not differ from that of the ‘water' group (145.30±13.71 g kg^−1^ per day, *n*=69). Daily ethanol quantity consumed reached a stable level of 10.52±0.30 g kg^−1^ per day (*n*=86) when free choice was given between water and 10% ethanol (Supplementary Figure S1 and Supplementary Table S1). This amount was in the same range as that reported in previous studies under the same conditions.^2^ These concentrations were sufficient to yield blood ethanol levels near 50–70 mg per 100 ml during the active drinking phase, that is, the dark phase of the circadian cycle.^12^ However, when measured 3 h after the light-on period, no more blood ethanol could be detected, as already described.^2^

### Methylation profile of the *Bdnf* gene after chronic and voluntary ethanol consumption

We first analyzed mRNA expression of three enzymes involved in methylation of cytosine at the level of the CG dinucleotide within the CpG island: DNA methyltransferase 1, implicated in the maintenance of the methylation profile during cell division, and DNA methyltransferases 3a and 3b, involved in *de novo* methylation. As shown in Table 1, reverse transcription and quantitative PCR analysis indicated that ethanol consumption led to a significant reduction in *Dnmt3b* mRNA expression (*F*_(5,38)_=4.895, *P*<0.05, one-way ANOVA with the Bonferroni multiple comparison test), whereas neither that of *Dnmt1* nor that of *Dnmt3a* was significantly modified after ethanol consumption (*F*_(5,38)_=4.895, *P*>0.05 and *F*_(5,38)_=4.895, *P*>0.05, respectively, one-way ANOVA with the Bonferroni multiple comparison test).

The analysis of various CpG island methylation within the *Bdnf* gene was performed using methylated DNA immunoprecipitation after laser microdissection of the dentate gyrus (DG) and the Ammon's horn 1 and 3 (CA1 and CA3) subfields of the hippocampus. A two-way ANOVA analysis showed a significant effect of ethanol consumption on DNA methylation in both CA1 and CA3 (*F*_(1,73)_=21.780, *P*<0.0001 and *F*_(1,84)_=6.137, *P*=0.0152, respectively) but not in the DG (*F*_(1,84)_=0.440, *P*=0.5089) and no effect on island methylation (CA1, *F*_(6,73)_=0.483, *P*=0.819; CA3, *F*_6,84)_=0.302, *P*=0.934; and DG, *F*_(6,84)_=0.513, *P*=0.797) and no interaction between factors (CA1, *F*_(6,73)_=0.483, *P=*0.819; CA3, *F*_(6,84)_=0.302, *P=*0.934; and DG, *F*_(6, 84)_=0.513; *P*=0.797; Table 2).

### Effects of chronic and voluntary ethanol intake on hippocampal BDNF TrkB signaling

**Figure 1:**
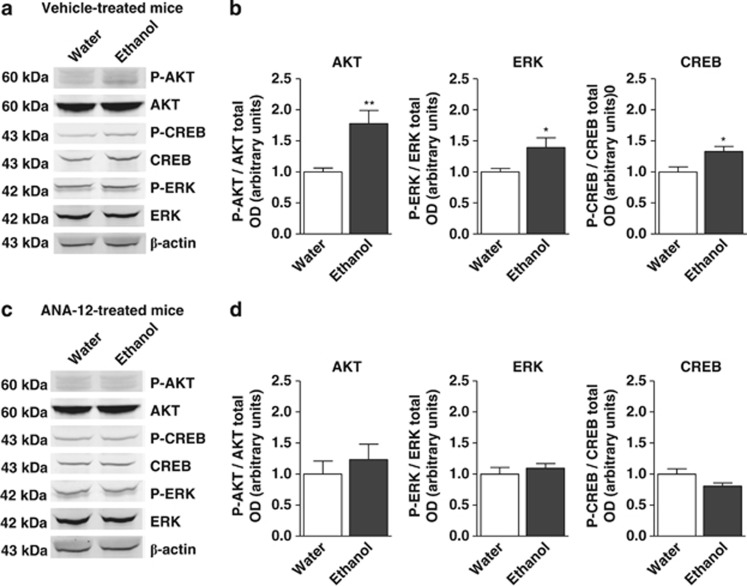
Effects of ethanol intake and ANA-12 on hippocampal brain-derived neurotrophic factor (BDNF) cellular signaling pathway. The effects of chronic ethanol intake and ANA-12 treatment, a BDNF TrkB receptor antagonist (0.5 mg kg^−1^ intraperitoneal/once a day), on BDNF signaling pathways were measured by immunoblotting. Western blot analyses of AKT and phospho (P)-AKT, ERK and P-ERK, and CREB and P-CREB were performed on hippocampal extracts of ‘water' and ‘ethanol' vehicle-treated (**a**, **b**) and ANA-12-treated mice (**c**, **d**). Quantifications of the western blots for P-AKT, P-ERK and P-CREB were expressed on the total level of proteins (**b**, **d**). Each bar is the mean±s.e.m. of *n*=6 (water) and *n*=9 (ethanol) mice; *P<0.05, **P<0.01, unpaired *t*-test.

To assess the effect of ethanol on the cellular signaling cascades downstream to BDNF TrkB receptors, we analyzed the phosphorylation of three proteins, AKT, ERK and CREB, involved in the regulation of proliferation, differentiation and survival in the neurogenesis process using western blot. As shown in Figure 1, chronic and voluntary ethanol intake stimulated the phosphorylation of AKT, ERK and CREB proteins in the hippocampus (*P*=0.008, 0.045 and 0.015, respectively, unpaired *t*-test; Figure 1b). To determine whether TrkB receptor activation was specifically involved in the ethanol effect, a similar experiment was conducted in mice that had been treated during the whole 10% ethanol intake period, with ANA-12, a TrkB receptor antagonist, which has already been reported^2^ not affecting either ethanol or water consumption. In this condition, the increase in AKT, ERK and CREB phosphorylation induced by ethanol was not observed anymore (*P*=0.546, 0.476 and 0.055, respectively, unpaired *t*-test; Figures 1c and d).

### Functional investigations

To determine whether the changes observed in cellular plasticity induced by chronic ethanol consumption in C57BL/6J mice could impact functional plasticity, we performed a series of electrophysiological and behavioral investigations.

#### Effects of chronic and voluntary ethanol intake on hippocampal LTP

LTP experiments were conducted in the CA1 region of hippocampal slices from ‘ethanol' and ‘water' mice.

**Figure 2:**
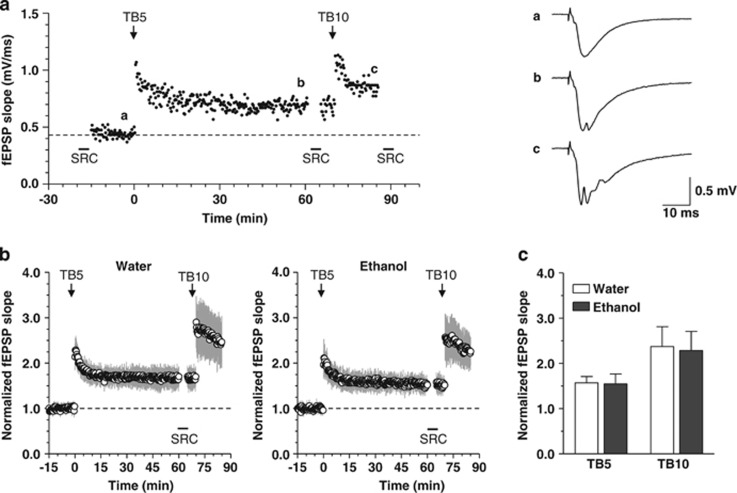
Effects of ethanol intake on long-term potentiation (LTP) in the CA1 region of the hippocampus. (**a**, left) Illustration of a representative experiment of LTP recording. Time course of changes in field excitatory postsynaptic potentials (fEPSPs) induced by TB5 and TB10 stimulation and recorded from the CA1 region of a slice taken from a ‘water' mouse. After collection of a stimulus–response curve (SRC, not shown) at the time indicated by the black square, the stimulation strength of the test pulse was set to produce a synaptic response with initial slope equal to ~30% of the maximum and baseline responses were collected for 15 min. TB5 and TB10 stimuli were delivered at time points indicated by arrows. TB5 stimulation-induced LTP was followed for 60 min before a second SRC was collected. Then, test stimuli were delivered for 5 min before applying TB10 stimulation that was followed for a further 15 min. (**a**, right) fEPSP responses recorded at time points indicated by corresponding letters in left graph. Traces are averages of 21 consecutive responses (5 min) recorded at baseline (**a**) and after TB5 (**b**), and of 9 responses (2 min) after TB10 (**c**). Upward deflections appearing in the traces **b** and **c** are back-propagating action potentials generated in the somatic region of pyramidal neurons after LTP induction. Calibration bars apply to all traces. (**b**) Graphs show the time courses of fEPSP slope changes induced by TB5 and TB10 stimuli in mice drinking tap water (‘water', left graph, *n*=19) or ethanol (‘ethanol', right graph, *n*=15). Symbols represent the mean±s.d. of fEPSP slope in individual experiments, normalized to the last 5 min before TB5 stimulation delivered at time zero. (**c**) Comparison of the normalized fEPSP slope after TB5 and TB10 showed no statistically significant difference between ‘water' and ‘ethanol' mice. Each bar is the mean±s.d. of *n*=19 (water) and *n*=15 (ethanol) mice.

Figure 2a illustrates a typical experiment in which synaptic LTP was induced by theta-burst stimulation delivered to the Schaffer collateral/commissural fibers and responses were recorded in the stratum radiatum from the dendritic region of CA1 pyramidal neurons. After induction of LTP by TB5 stimulation, responses to test stimuli were monitored for 1 h; then, a second theta-burst stimulus (TB10) was delivered to produce a near-maximal increase in synaptic responses. As shown in Figure 2b, TB5 stimulation induced a significant facilitation of synaptic responses in both ‘water' and ‘ethanol' mice, as shown by the increase in the fEPSP slope that persisted for 1 h (‘water', 57±0.04%, *P*<0.0001, *n*=19; ‘ethanol', 54±0.04%, *P*<0.0001, *n*=15; Wilcoxon test; Figure 2c). The increases in the fEPSP slope obtained after TB5 or TB10 in ‘ethanol' mice were not statistically different from those measured in ‘water' mice (*P*=0.687 and 0.325, respectively, Mann–Whitney test; Figure 2c).

#### Contextual fear memory and object recognition memory after chronic ethanol consumption

**Figure 3:**
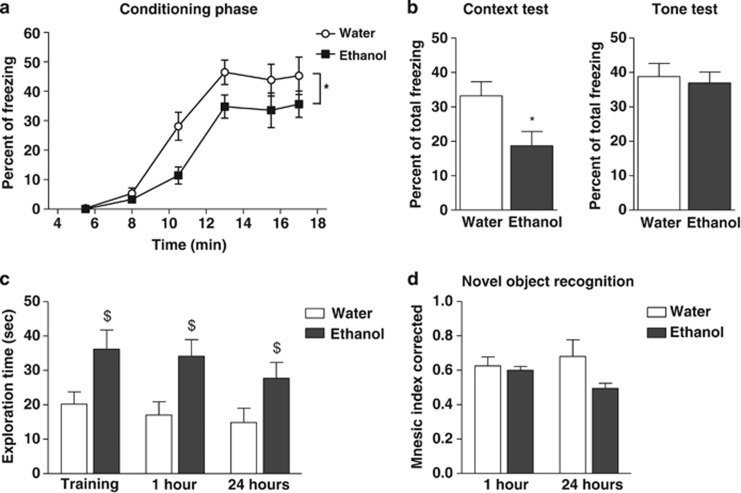
Effects of ethanol intake on contextual fear memory and object recognition. (**a**, **b**) Cued and contextual fear memory was evaluated using the fear conditioning test. (**a**) Freezing was measured for each conditioning stimulus given to the mice followed by an intertrial interval. Points represent percent of freezing in function of the time. Each point is the mean±s.e.m. of *n*=6 (water) and *n*=9 (ethanol) mice; *P<0.05, repeated measures two-way analysis of variance (ANOVA). (**b**) Percent of total freezing during the contextual test and the tone test is represented. Each bar is the mean±s.e.m. of *n*=6 (water) and *n*=9 (ethanol) mice; *P<0.05, Student's *t*-test. (**c**, **d**) Mnesic index was evaluated in the novel object recognition test. (**c**) Time spent exploring the objects was quantified during the acquisition and within the testing sessions 1 and 24 h post trial. Each bar is the mean±s.e.m. of *n*=5 (water) and *n*=8 (ethanol) mice; ^$^*P<*0.05, repeated measures two-way ANOVA. (**d**) Mice recognition performances were represented as the corrected mnesic index for the new object. Each bar is the mean±s.e.m. of *n*=5 (water) and *n*=8 (‘ethanol') mice; *P<0.05, Bonferroni's multiple comparison test.

During the conditioning phase, a two-way ANOVA revealed a significant effect of ethanol on freezing, an effect of time and no interaction (*F*_(1,60)_=7.620, *P*=0.017; *F*_(5,60)_=52.230, *P*<0.0001; and *F*_(5,60)_=1.434, *P*=0.225, respectively, repeated measures two-way ANOVA; Figure 3a). In the contextual fear conditioning test, ‘ethanol' mice displayed less freezing than ‘water' mice (*P*=0.031; Student's *t*-test; Figure 3b, left). However, during the tone test to assess cued conditioned fear, the percentage of freezing recorded in ‘ethanol' and ‘water' mice did not differ (*P*=0.956, Student's *t*-test; Figure 3b, right).

To assess whether the decrease in contextual fear was due or not to an anxiolytic effect of ethanol, anxiety-like behaviors were analyzed using the elevated plus maze and the open field. Neither the number of entries nor the time spent in the open arms was changed in ‘ethanol' compared with ‘water' mice (*P*=0.196 and 0.977, respectively, Student's *t*-test; Supplementary Figure S2A). Similarly, in the open field, the ambulation and the time spent in the central area of the open field were not modified in ‘ethanol' compared with ‘water' mice (*P=*0.260 and 0.731, respectively, Student's *t*-test; Supplementary Figure S2B).

As the above data suggested that ethanol could specifically impact hippocampus-related memory, and we then performed the novel object recognition task test at the end of the 21-day free-choice ethanol session. A two-way ANOVA on the time spent exploring objects during the acquisition and the testing sessions (1 and 24 h after the training) showed an effect of ethanol, an effect of time and no interaction between these two variables (*F*_(1,22)_=5.292, *P*<0.05; *F*_(2,22)_=5.069, *P*<0.05; and *F*_(2,22)_=0.489, *P>*0.05, respectively, repeated measures two-way ANOVA; Figure 3c). A two-way ANOVA on the corrected mnesic index during the testing sessions (1 and 24 h) showed no effect of sessions, a significant effect of ethanol and no interaction between theses variables (*F*_(1,11)_=0.244, *P*>0.05; *F*_(1,11)_=4.872, *P*<0.05; and *F*_(1,11)_=3.242, *P*>0.05, respectively, repeated measures two-way ANOVA; Figure 3d).

#### Effects of chronic ethanol intake on spatial memory and perseveration

**Figure 4:**
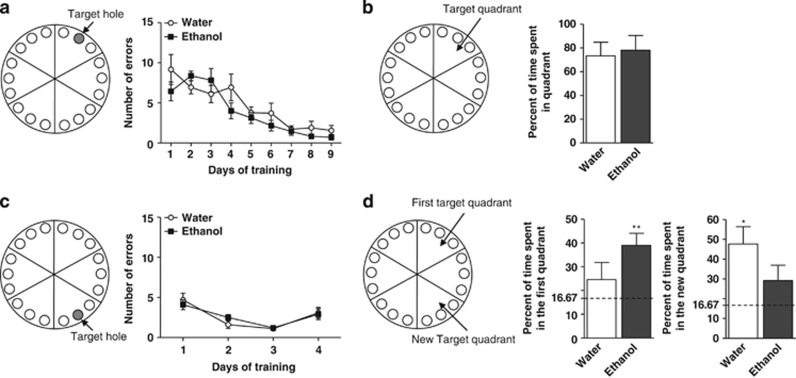
Effects of ethanol intake on spatial memory and cognitive flexibility. (**a**, left) Schematic representation of the Barnes test. Gray circle represents the escape box localized beneath the hole. (**a**, right) Mice were trained to learn the location of the escape box for 9 days with 2 sessions per day and the number of errors to reach the box (spatial memory) was recorded and plotted in function of the days. Each point is the mean±s.e.m. of *n*=10 (water) and *n*=9 (ethanol). (**b**, left) The circular platform was arbitrarily divided into six quadrants and the quadrant that contained the target hole was called the target quadrant. The escape box was removed and the mice were placed on the platform and allowed to explore during 30 s. (**b**, right) Percentage of time spent in the target quadrant during 30 s of the probe test. Each bar is the mean±s.e.m. of *n*=8 (water) and *n*=7 (ethanol) mice. (**c**, left) Reversal learning. The location of the escape box was moved 120° from the original location. (**c**, right) The reversal learning was monitored during 4 days with 3 sessions per day and the number of errors to reach the new target hole was recorded. Each point is the mean±s.e.m. of *n*=8 (water) and *n*=7 (ethanol). (**d**, left) The escape box was removed for the reversal probe test. Mice were placed on the platform and allowed to explore. (**d**, right) The percent of time spent in the new quadrant and in the first quadrant containing the original location of the escape box was analyzed during 30 s . Each bar is the mean±s.e.m. of *n*=8 (water) and *n*=6 (ethanol) mice, compared with the hypothetical value of 16.67; *P<0.05, **P<0.01, one-sample *t*-test.

We further explored spatial capacities and perseveration to return to a learned location, which are both hippocampal-dependent cognitive tasks, using the Barnes maze. ‘Ethanol' and ‘water' mice were trained to learn the location of an escape box during 9 days with 2 sessions per day (Figure 4a, left). The number of errors to reach the escape box was quantified for each session as presented in Figure 4a (right). A two-way ANOVA revealed no effect of ethanol intake on the number of errors, a significant effect of time and no interaction between factors (*F*_(1,149)_=3.807, *P*=0.053; *F*_(8,149)_=13.950, *P*<0.0001; and *F*_(8,149)_=1.534, *P*=0.150, respectively, two-way ANOVA). There were no changes between groups in the primary latency to reach the target (Supplementary Figure S3A) and in the distance traveled before reaching the escape box (Supplementary Figure S3B). The quadrant test consisted in removing the escape box, and the time spent in the quadrant previously containing the escape box was quantified (Figure 4b, left). The percentage of time spent in the target quadrant was not different between ‘ethanol' and ‘water' mice (*P*=0.463, Student's *t*-test; Figure 4b, right).

To assess cognitive flexibility, the position of the escape box was moved 120° from its original location and the mice were trained again in these conditions (Figure 4c, left). Training consisted of 4 days with 3 sessions per day. The number of errors to reach the new hole is represented in Figure 4c (right). A two-way ANOVA revealed no effect of chronic ethanol consumption, a significant effect of time and no interaction between variables (*F*_(1,52)_=0.003, *P*=0.960; *F*_(3,52)_=12.78, *P*<0.0001; and *F*_(3,52)_=0.449, *P*=0.719, respectively, two-way ANOVA). Here again there were no changes between groups in the primary latency to reach the target (Supplementary Figure S3C) and in the distance traveled to find the escape box (Supplementary Figure S3D).

The reversal probe test consisted of measuring (1) the time spent in the new target quadrant and (2) the time spent in the first target quadrant after reversal learning. The latter is representative of mice perseveration to return to the initial target (Figure 4d, left). The theoretical random percentage of time spent in each quadrant is 16.67%, as the platform is divided into six identical quadrants. Comparison of means from each group with the theoretical value of 16.67% showed that, as expected, the ‘water' group had statistically higher explorations in the new target quadrant (*P=*0.016, one-sample *t*-test). In contrast, the ‘ethanol' group did not differ from random exploration (*P=*0.166, one-sample *t*-test; Figure 4d, right). Furthermore, while the time spent in the initial quadrant of the ‘water' mice did not differ from random exploration after reversal learning (*P=*0.315, one-sample *t*-test), that of ‘ethanol' mice was significantly increased (*P=*0.005, one-sample *t*-test; Figure 4d, center), suggesting a perseveration behavior.

Finally, basal locomotor activity and motor coordination were analyzed to assess whether the above modifications were related to an effect of alcohol on these parameters. A two-way ANOVA analysis showed an effect of time on spontaneous locomotor activity in the actimeter, while no effect of chronic ethanol intake and no interaction between variables (*F*_(11,143)_=48.950, *P*<0.0001; *F*_(1,143)_=0.302, *P*=0.592; and *F*_(11,143)_=1.364, *P*=0.196, respectively, repeated measures two-way ANOVA; Supplementary Figure S4A). The numbers of rearing and beam crossing were not affected by chronic ethanol consumption (*P*=0.961 and 0.795, respectively, Student's *t*-test; Supplementary Figure S4A).

In the accelerating rotarod, motor coordination was not affected in ‘ethanol' compared with ‘water' mice (trials, *F*_(2,32)_=12.38, *P*=0.0001; ethanol, *F*_(1,32)_=0.462, *P=*0.506; and interaction, *F*_(2,32)_=0.174, *P*=0.841, repeated measures two-way ANOVA; Supplementary Figure S4B).

## Discussion

The present data showed that chronic free-choice ethanol downregulated *Bdnf* gene DNA methylation within the CA1 and CA3 subfields of the hippocampus. These data completed those previously published, and which suggested post-translational histone modifications and an increase in both *Bdnf* mRNA and BDNF protein using the same ethanol intake paradigm.^2^ In addition, we showed that ethanol-induced BDNF expression through epigenetic modifications upregulated intracellular BDNF signaling pathways. The involvement of BDNF TrkB receptors in this effect was demonstrated by the absence of ethanol impact in presence of the selective TrkB receptor antagonist ANA-12 (ref. 14). Although those modifications in cellular plasticity did not result in LTP modification in the CA1 region, they were nevertheless associated with deficits in learning and memory performances in both the contextual fear and the novel object recognition paradigms. In addition, using the Barnes test, ‘ethanol' mice showed perseveration behavior, without any alteration in spatial learning and memory. Altogether, these data suggest that chronic ethanol intake led to alterations in cognitive performances, which implicated hippocampal processing, and that the upregulation in BDNF gene expression and signaling pathway was probably a reactive process to counteract ethanol-induced behavioral deficits.

We, as well as others,^17^ previously reported that low and chronic ethanol consumption in C57BL/6J mice surprisingly increases hippocampal neurogenesis.^2^ This ethanol-induced plasticity was shown to occur through histone post-translational modifications.^2^ Here we further characterized epigenetic modifications induced by chronic ethanol by studying DNA methylation in C57BL/6J mice. We first showed that between the three active DNA cytosine methyltransferases identified in human and mouse, free-choice ethanol downregulated *Dnmt3b* involved in *de novo* gene methylation. Although DNMT3a and DNMT3b are extremely similar in structure and function,^18^ some data suggested that DNMT3b may be specifically involved for the early phase of neurogenesis,^19^ which was shown to be modified under ethanol consumption.^2^

In addition, ethanol reduced *Bdnf* methylation in Ammon's horn CA1 and CA3 hippocampal areas. Interestingly, several studies have reported methylation modulation under ethanol intake. Indeed, Ponomarev *et al.*^20^ recently reported that ethanol could impact gene- and cell-specific DNA methylation. In addition, ethanol has been found to impair the synthesis of methyl donor group *S*-adenosylmethionine by inhibiting the enzymes that regulates its production^21^ and in consequence to impair DNA methylation, which is directly affected by *S*-adenosylmethionine levels. Furthermore, chronic ethanol consumption in human has been shown to induce a global DNA hypomethylation and to alter DNA methyltransferase expression.^22^ All these data support our results suggesting that free ethanol consumption can promote the expression of BDNF^2^ by reducing *Bdnf* gene methylation through modulation of enzymes that control the synthesis of the methyl donor group and also by inhibiting enzymes that add methyl group on CpG sites, in addition to the post-translational histone modifications previously observed.^2^

To verify that the increased expression of BDNF observed after free-choice ethanol,^2^ linked to epigenetic modifications, could induce functional cellular regulations, we assessed various BDNF signaling pathways. Chronic ethanol consumption enhanced P-ERK/ERK and P-AKT/AKT ratios leading to an increase in CREB activation. This effect appears to be associated with BDNF TrkB activation as ethanol no longer had effect under ANA-12, a selective TrkB receptor inhibitor,^14^ which, by itself, did not modify ethanol intake. Interestingly, systemic administration of ANA-12 to mice was reported to decrease TrkB activity in the brain without affecting neuronal survival, while it was shown to prevent the hippocampal neurogenic effect of BDNF,^2, 14^ and to reduce anxio-depressive behavior in mice (Boulle *et al.*, in revision). Consistently, ethanol-related behaviors have previously been associated with changes in the phosphorylation level of several BDNF signaling pathways, including CREB, ERK and AKT.^8, 23, 24, 25^ These signaling pathways have also been shown to be involved in the regulation of hippocampal plasticity, neurogenesis and BDNF stimulation.^26^ It is noteworthy that AKT signaling is involved in cell survival process through the stimulation of the anti-apoptotic protein Bcl-2, which is also increased by free-choice ethanol in these mice^2^ probably through the positive action of *N*-methyl-d-aspartate, as proposed by Bhave *et al.*^27^ Nonetheless, these results are in sharp contrast to those reporting an inhibitory effect of chronic or acute ethanol exposure on the phosphorylation of these proteins.^28, 29, 30^ This discrepancy was probably related to the ethanol peak concentration reached in brain following different protocols of ethanol administration. Indeed, forced administration of high doses of ethanol leads to blood alcohol level reaching 200 mg%, while the free-choice ethanol intake protocol we applied here leads to a blood alcohol level of only 50 mg% (ref. 2).

Whether these modifications in BDNF expression and cellular plasticity could result in functional adaptations was then addressed. Preclinical studies in the past two decades have demonstrated the role of BDNF, TrkB and TrkC receptors in modulating synaptic plasticity.^31, 32, 33, 34^ Hippocampal LTP is widely considered as a cellular paradigm of synaptic plasticity correlated with learning and memory,^35^ and as a requirement for hippocampal processing and encoding of spatial and emotional memory traces.^36^ It involves Schaffer collateral/commissural pathway plasticity.^37^ The effects of ethanol on LTP have been extensively studied. A reduction in the magnitude of LTP has been consistently reported in the hippocampus of rats chronically treated with high ethanol doses after either a long-term (28 weeks) treatment,^38^ an intermittent vapor exposure mimicking human binge alcohol,^39^ or a 12-week chronic, forced, ingestion of liquid diet.^40^ Surprisingly, we could not detect any significant modification in LTP magnitude in ‘ethanol' mice compared with ‘water' mice, indicating that the cellular mechanisms of synaptic plasticity underlying CA1 hippocampal encoding in the CA1 region of the hippocampus were not disrupted or enhanced by chronic ethanol intake for 21 days.

However, the functional connectivity of the hippocampus underlying spatial, contextual and emotional memories differentially involves various brain regions whose activity, in turn, contributes to hippocampal processing, memory acquisition and performance.^41, 42, 43^ Chronic ethanol has been found to affect several neurotransmitter systems and hormones that control neuron excitability in cortical and subcortical areas implicated in memory processes.^44, 45, 46, 47^ In the behaving animal, functional changes in neuronal processing produced by chronic ethanol in any of these brain areas could contribute to lessening in the corresponding memory tasks, without requiring an impairment of cellular mechanisms of synaptic plasticity. This may explain the selective impairment observed in some, but not all, of the hippocampus-dependent behavioral responses.

The preservation of synaptic plasticity in the hippocampal CA1 region appears to be consistent with the absence of impairment in spatial learning and memory observed in the Barnes maze test, although ethanol was previously shown to interfere with spatial performances in both rodents and humans.^48, 49^

It is conceivable that the impairment of synaptic plasticity regularly reported under different regimens of chronic ethanol administration are mitigated by the changes in plasticity observed at the BNDF complex level in our conditions of chronic moderate ethanol intake. In other terms, increased AKT, ERK and CREB in the hippocampus could be a reactive compensation to limit the deleterious effects of ethanol in mnesic functions.

Conversely, in the fear conditioning paradigm, chronic ethanol consumption decreased the speed of contextual fear acquisition. This effect was not associated with an anxiolytic-like effect of alcohol and is in accordance with studies showing that ethanol exposure impairs memory acquisition and performances.^50^ Furthermore, although freezing in ‘ethanol' mice was decreased in the contextual fear test, it was not changed in the auditory cue fear test, further arguing that in our conditions of alcohol intake, mice can express an equal amount of fear. The deficit observed in the fear conditioning in the alcohol group could result from neuronal alterations in several brain regions, such as the amygdala and the hippocampus, which have key roles in both cue and contextual fear conditioning.^51, 52^ However, an involvement of the amygdala to explain the present data seems unlikely because there is no memory deficit in the cue fear conditioning test. Several lines of evidence indicate that contextual, but not cue-induced, fear is hippocampus dependent,^53^ suggesting that alcohol exposure promoted a deficit in hippocampus-related memory. These results are in accordance, at least in part, with other data showing that acute high doses of ethanol injection impairs contextual but also cue fear C57BL/6J conditioning.^54, 55^ A study using acute ethanol injection in rats also showed that ethanol differentially altered contextual versus cue fear conditioning, depending on the dose injected: whereas a low dose of ethanol seems to impair contextual fear only, a high dose deteriorates both contextual and cue fear conditioning.^56^

Whether this low intake of ethanol could also impact mnesic functions was also investigated in the novel object recognition paradigm. In this test, the performances of ‘ethanol' mice for the object recognition were lower than that of ‘water' mice.^57^ This decrease in cognitive function was rather modest, but the exploration behavior of ‘ethanol' mice was significantly higher compared with ‘water' mice, whatever the sessions, training or tests performed after the 1- and the 24-h delay, suggesting an increase in perseveration to explore objects. This perseveration behavior was also observed in the Barnes maze during the quadrant test after reversal learning. Indeed, ‘ethanol' mice did not completely extinct their approach toward the initial target hole, in contrast to the ‘water' mice. However, there was no indication that cognitive flexibility itself, that is, the capacity to learn a new target was not modified by ethanol. Interestingly, under these conditions of ethanol administration, neurogenesis is stimulated,^2^ while a recent study also demonstrated behavioral perseveration in the Morris Water maze in correlation with stimulated hippocampal neurogenesis in mice.^58^ However, other hypothesis may also account for the increase in perseveration behavior after ethanol consumption such as an alteration in LTD or a depotentiation at the CA1 level.^34, 59, 60^

In conclusion, we provide the evidence that ethanol-regulated intracellular signaling pathways involving AKT, ERK and CREB, through the increase in BDNF expression occurs after DNA methylation, as demonstrated in the present study, and chromatin remodeling modifications as we previously demonstrated.^2^ However, this chronic and moderate ethanol consumption led to impairments in learning, memory and behavioral perseveration that are functions controlled among others, by the hippocampus. These deficits were not associated with changes in hippocampal LTP, suggesting that mechanisms other than hippocampal synaptic plasticity as modeled by LTP were involved in the regulation of mnesic performances, or that the chromatin remodeling leading to BDNF signaling upregulation could reverse some synaptic plasticity dysregulations but were not enough to counteract all the cognitive deficits induced by ethanol. In addition, the cognitive deficits observed here with ethanol could be the consequences of alterations in cortical structures such as the enthorinal or the prefrontal cortex,^61^ or the cholinergic tonus.^62^

Our data suggest that in conditions of chronic and moderate ethanol intake, the chromatin remodeling leading to BDNF signaling upregulation is an adaptive process engaged via epigenetic regulations, to limit the cognitive impairments generated by ethanol.

## Figures and Tables

**Table 1:** Effects of chronic ethanol intake on hippocampal levels of mRNAs encoding DNA methyltransferases

*Gene*	*Water*	*Ethanol*	*Percent over water*	P*-value*
*Dnmt1*	1.014±0.090 (5)	0.918±0.050 (9)	−9.47	NS
*Dnmt3a*	1.005±0.042 (6)	1.167±0.059 (9)	+16.12	NS
*Dnmt3b*	1.018±0.087 (6)	0.795±0.043 (9)	−21.91	*

Abbreviations: ANOVA, analysis of variance; NS, not significant.

*P<0.05, significantly different in ‘ethanol' versus ‘water' mice.

mRNA levels were measured by real-time reverse transcription and quantitative PCR in the hippocampus of ‘water' and ‘ethanol' mice. Results (mean±s.e.m. of (*n*) mice) are expressed as arbitrary units after normalization to the endogenous reference genes encoding *Hprt* and *β-actin*. One-way ANOVA followed by Bonferroni's multiple comparison test.

**Table 2:** Effects of chronic ethanol intake on the *Bdnf* gene methylation in the dentate gyrus, CA1 and CA3 subfields of the hippocampus

Bdnf *CpG Islands*	*Water*	*Ethanol*
*Dentate gyrus*		
I	1.000±0.006 (6)	1.006±0.010 (8)
II	1.000±0.009 (6)	1.001±0.007 (8)
III	1.000±0.012 (6)	0.987±0.006 (8)
IV	1.000±0.010 (6)	1.006±0.005 (8)
V	1.000±0.010 (6)	1.004±0.007 (8)
VI	1.000±0.009 (6)	1.002±0.006 (8)
VII	1.000±0.008 (6)	1.014±0.003 (8)
*Gapdh*	1.000±0.008 (6)	1.011±0.006 (8)

Bdnf *CpG Islands*	*Water*	Ethanol*
*CA1*		
I	1.000±0.013 (5)	0.976±0.006 (8)
II	1.000±0.014 (5)	0.969±0.006 (8)
III	1.000±0.014 (5)	0.977±0.004 (8)
IV	1.000±0.012 (5)	0.974±0.009 (8)
V	1.000±0.013 (5)	0.956±0.008 (8)
VI	1.000±0.009 (5)	0.985±0.008 (8)
VII	1.000±0.015 (5)	0.985±0.014 (8)
*Gapdh*	1.000±0.014 (5)	0.991±0.011 (8)

Bdnf *CpG Islands*	*Water*	Ethanol**
*CA3*		
I	1.000±0.009 (6)	0.991±0.008 (8)
II	1.000±0.010 (6)	0.985±0.008 (8)
III	1.000±0.005 (6)	0.997±0.009 (8)
IV	1.000±0.005 (6)	0.998±0.007 (8)
V	1.000±0.007 (6)	0.988±0.008 (8)
VI	1.000±0.010 (6)	0.984±0.008 (8)
VII	1.000±0.006 (6)	0.982±0.008 (8)
*Gapdh*	1.000±0.006 (6)	0.997±0.007 (8)

*P<0.05.

**P<0.0001.

Methylation enrichment levels on the different *Bdnf* CpG islands were measured by real-time quantitative PCR of the immunoprecipitated DNA with a specific antibody against 5-methylcytidine in the three subfields of hippocampus in ‘water' and ‘ethanol' mice. Results (mean±s.e.m. of (*n*) mice) are expressed as arbitrary units after normalization of the immunoprecipitated DNA/input DNA ratio to that of the control group of ‘water' mice for each CpG island. A two-way analysis of variance showed an effect of ethanol on the methylation of *Bdnf* CpG islands in CA1 and CA3 subfields.
