# Supplementary material for: Brain plasticity and cognitive functions after ethanol consumption in C57BL/6J mice
Source: Transl Psychiatry. 2015 Dec 15;5(12):e696–. doi: 10.1038/tp.2015.183 (PMC5068583; doi:10.1038/tp.2015.183)
Supplement: Supplementary Materials and Methods [file tp2015183x1.docx]

**Supplementary Materials and Methods**

**Free-choice ethanol paradigm**

Mice were housed singly in standard laboratory cages (Macrolon type 2, 22 x16 x14 cm) equipped with four tubes (20 mL), one containing tap water and the other three filled with ethanol dilutions in water for “ethanol” mice, while all tubes were filled with tap water for “water” mice. Previous data (Tordoff et al. 2003; Kelai et al. 2008) indicated that, in these conditions, the amount of ethanol ingested daily was slightly but regularly larger than the amount ingested when only one bottle of ethanol was offered versus one bottle of tap water. Animals had continuous free access to the drinking tips of tubes. “Ethanol” mice were exposed progressively to increasing concentrations of ethanol (0–10% ethanol in water within 21 days) under the free-choice procedure described by Kelaï *et al*. (Figure S1). Briefly, mice were offered 3% ethanol (v/v) versus water for 4 days, then 6% ethanol versus water for the next 4 days, and finally animals had access to 10% ethanol versus water for the subsequent 12 days (stabilization period). Food (standard mouse pellets A03; UAR, Strasbourg, France) was given ad libitum. Absolute amounts of ethanol (as grams) consumed and total fluid (grams of ethanol solution plus tap water) intake/kg body weight/24 h were determined for each mouse. Mice were sacrificed between 10:00 and 12:00 am on day 21, trunk blood samples were taken and blood ethanol concentrations were determined using a chemical analyser (Dimension^®^ Xpand^®^ Plus, Dade Behring, Paris, France).

**Reverse transcription (RT) and quantitative PCR**

For these assays, total mRNA was extracted using NucleoSpin^®^ RNA II kit (Macherey-Nagel, Hoerdt, France) following manufacturer's instructions. RT was performed using High Capacity cDNA Reverse Transcription kit (Applied Biosystems, Courtaboeuf, France) with the following protocol: 25°C, 10 min; 37°C, 2 h; 85°C, 5 s. The amplification reactions were performed with KAPA SYBR^®^ FAST qPCR Kit (KAPABIOSYSTEMS, Clinisciences, Nanterre, France) using the 7300 Real Time PCR System (Applied Biosystems) with the following cycling protocol: 95°C, 3 min; followed by 40 cycles of 95°C, 15 s; 60°C, 30 s; 72°C, 30 s.

**Laser-assisted microdissection**

Coronal sections of 40 µm thickness were collected at the hippocampus level using a microtome (HM450, MM France, Francheville, France), between the planes “Bregma – 1.22 mm” and “ Bregma – 2.30 mm” (Franklin and Paxinos, 1997). Sections were mounted on polyethylene naphthalate membrane slides (Zeiss, Le Pecq, France). After a 3 min dry, sections were stained with Hematoxylin (15 sec) then rinsed in distilled water (2-3 sec), stained with Eosin (25 sec), successively deshydrated with 50%, 70% and absolute ethanol (1 min), and allowed to dry for 3 min. Laser-assisted microdissection was performed on a PALM Microbeam Zeiss instrument with a PALMRobo software (Zeiss). Micro-dissected tissues from the hippocampus (dentate gyrus [DG], Ammon’s horn 1 and 3 [CA1 and CA3]) were suspended into a lysis buffer from the NucleoSpin® Tissue kit (Macherey Nagel, Hoerdt, France).

**Methylated DNA immunoprecipitation (MeDIP)**

gDNA was diluted in Tris-EDTA buffer (10 mM Tris-HCl pH 7.5, 1 mM EDTA) and sonicated using a Bioruptor UCD-200 (Diagenode, Liège, Belgium) with the following program: intensity High, 30 s ON/30 s OFF, 5 min, 4°C. The DNA solution was heat-denaturated for 10 min at 95°C, immediately cool on ice for 5 min and 100 µL of 5X Immunoprecipitation buffer (IP) (50 mM phosphate buffer pH 7.0, 700 mM NaCl, 0.25% Triton X-100) was added. The resulting DNA solution was pre-cleared with 30 µL of magnetic beads, previously blocked with 0.5% BSA, for 1 hour at 4°C. 80 µL of supernatants were saved as “INPUT” for later normalization and stored at -20°C until protein digestion. Methylated DNA was incubated overnight at 4°C with antibody against 5-Methylcytidine (BI-MECY, Eurogentec, Angers, France). DNA-antibody complexes were immunoprecipitated by adding 30 µL of magnetic beads and incubated 2 hours at 4°C, and then the beads were washed three times with 1 mL of 1X IP buffer for 5 min at 4°C. The beads were resuspended in 250 µL of digestion buffer (50 mM Tris-HCl pH 8.0, 10 mM EDTA, 0.5% SDS) and the 80 µL of INPUT in 170 µL of the same buffer. 30 µg of proteinase K was added to both beads and INPUT and incubated overnight at 56°C. DNA was extracted using a phenol / chloroform / isoamyllic alcohol method. DNA was then precipitated by adding 500 µL of absolute ethanol, 20 µL 5M NaCl and 20 µg glycogen and incubated 2 hours at -20°C. Pellets were washed with 70 % ethanol and suspended in 30 µL of water.

**Immunoblotting**

Total proteins were extracted using a lysis buffer (HEPES 50 mM, NaCl 150 mM, 0.5% Triton X-100) containing freshly prepared protease and phosphatase inhibitors (PIC: Protease Inhibitor Cocktail, 12505200, Roche, Meylan, France; sodium orthovanadate 2 mM and sodium fluoride 2 mM). Samples were sonicated and centrifuged at 14,000 × g for 30 min at 4°C. Supernatants were removed and frozen at -20°C until western blotting experiments. The protein content in supernatants was measured using the BCA protein assay kit (Pierce Biotechnology, Thermo Fisher Scientific, Courtaboeuf France). Proteins (50 µg) were electrophoresed in duplicate in a 4-12% Bis-Tris gel (Novex, Life Technologies, Saint Aubin, France), then electrically transferred from the gel to a nitrocellulose membrane (Novex, Life Technologies) using the iBlot System (Novex, Life Technologies) according to the manufacturer’s instructions. Membranes were saturated 1 h at room temperature with 5% BSA (w/v) in Tris-buffered Saline with 0.1% Tween-20 (TBST) then incubated overnight in TBST with 5% BSA with one of the primary antibodies listed in the Materials and Methods section. After extensive washing in TBST buffer, membranes were incubated with anti-rabbit IRdye 800CW-coupled IgG (Rockland Immunochemicals, Gilbertsville, PA) or anti-mouse IRdye 700DX-coupled IgG (Rockland Immunochemicals). Fluorescence of the immunoblots was analysed at 800 nm and 700 nm using an Odyssey infrared imager (Li-Cor, Lincoln, NE).

**Long-term potentiation (LTP) experiments**

*Preparation of hippocampal slices*

Mice were anesthetized with isoflurane and decapitated at the beginning of the light phase. Hippocampi were rapidly removed and placed in ice-cold artificial cerebrospinal fluid (ACSF), which contained the following (in mM): NaCl, 124; KCl, 2.75; NaH2PO4, 1.25; NaHCO3, 26; MgSO4, 1.3; CaCl2, 2; d-glucose 10. The solution was bubbled with a 95% O2/5% CO2 gas mixture (pH 7.4). Transversal hippocampal slices of 400 µm nominal thickness were cut with a McIlwain tissue chopper (Gomshall, U.K.). After discarding approximately 2 mm of the dorsal hippocampal pole, seven to nine slices were cut. All slices were kept in a multi-well incubation chamber for at least 1 h at room temperature until recording.

*Electrophysiological recordings*

Hippocampal slices were obtained as previously described (Mlinar *et al*., 2008). Before transferring to the recording chamber, a single slice was temporarily transferred to a Petri dish, where the area CA1 was disconnected from the area CA3 by a surgical cut. The slice was then placed on a nylon mesh, completely submerged in a recording chamber and continuously superfused (2 ml min^− 1^) with oxygenated ACSF at 32–33 °C. Slices were incubated for 15 min in the recording chamber before initiating electrical stimulation. Stimulation pulses (80 μs duration; 15 s interpulse interval), triggered by a PC controlled by pClamp 9 (Molecular Devices, Foster City, CA, US) were delivered by a stimulus isolation unit (DS2, Digitimer, Welwyn Garden City, U.K.) through a concentric bipolar electrode (FHC, Bowdoin, ME, US). Evoked potentials were recorded with glass electrodes filled with 150 mM NaCl (2–10 MΩ resistance) placed in the distal third of the *stratum radiatum* to record field excitatory postsynaptic potentials (fEPSP). The distance between the stimulating and recording electrodes was 300-400 μm. Recorded potentials were amplified with Neurolog NL 104 amplifiers (Digitimer), digitized with the sampling rate of 50 kHz (Digidata 1322A, Molecular Devices) and stored in a PC for off-line analysis. At the beginning of each experiment, a stimulus–response curve (SRC), obtained by gradually increasing stimulus intensity, was recorded. The fEPSP was determined as the slope of the initial falling phase of the response recorded in the *stratum radiatum* following the afferent volley (Johnston & Wu 1995) and measured by linear regression in the region between 30 and 70% of the fEPSP. For each input, the stimulus intensity of test pulses was set to evoke a fEPSP that had an initial slope of ~30% (range 25-35%) of the maximum obtained in the stimulus-response curve (SRC) and stimulus intensity was held constant throughout the remainder of the experiment.

### *LTP protocol*

Input pathway was stimulated every 15 s, at the stimulus intensity set to produce fEPSPs with the slope of ~30% of the maximal slope obtained from the stimulus–response relationship. After 15 min of stable baseline responses LTP was induced by theta-burst stimulation consisting of a single train of 5 bursts of 5 stimuli (100 Hz intraburst frequency, 5 Hz burst frequency; called TB5) and followed for 60 min. TB5 stimulation was chosen among the possible patterns of theta rhythm-based stimulations because it produces LTP of intermediate magnitude, thus allowing for detection of modulatory effects in both inhibitory and facilitatory directions. At the end of the experiment (60 min after TB5) a SRC was generated and, after a further 5 min recording at the test stimulus strength, the pathway was activated with TB10 stimulation (10 bursts of 5 stimuli, 100 Hz intraburst frequency, 5 Hz burst frequency) to evoke the maximally achievable potentiation in the preparation (Mlinar *et al*. 2014). The response was followed for 15 min and the last 2 min were used for measuring maximal potentiation that can be achieved in each slice. Steady-state values of potentiation produced by TB5 stimulation were obtained by averaging the values of the 21 consecutive responses recorded over the 5-min period between 55-60 min after TB5 stimulation. The maximally achievable potentiation was calculated by averaging the values of 9 responses over the 2-min period between 13-15 min after TB10 stimulation.

**Elevated Plus-Maze (EPM)**

The apparatus was elevated 50 cm above the floor and consisted of 2 opposite open arms (40 x 5 cm) crossing 2 closed arms (40 x 5 x 18 cm). Room lighting was kept at 150 lux. Mice were placed onto the central platform facing a closed arm and free to explore the apparatus for 5 min. The exploratory behavior of mice was automatically collected using a video tracking system (Viewpoint, Lyon, France). The number of entries and time spent in the open arms were used as an index of anxiety-like behavior.

**Open field test**

The apparatus consisted of four open boxes (50 x 50 cm) separated by white wood walls and equipped with an infrared floor for the measurement of exploratory behavior. The arena was subdivided into a central and a peripheral zone. Mice were placed in the open field boxes for 15 min under normal light conditions, and the exploratory behavior of the animals was automatically measured with a video tracking system (Viewpoint, Lyon, France). Both the time spent and the distance covered in the different zones were scored.

**Spontaneous locomotor activity**

Locomotor activity was measured using a computer-based photo beam apparatus. The actimetry box (area: 300 x 150 mm^2^, 180 mm height, with Plexiglas wall and grid floor) detected mouse movements by means of two infrared light beams. Mice were placed in the testing apparatus during 1 hour. Both ambulations, corresponding to the number of squares crossed in the periphery and the center of the actimeter, and rearings, corresponding to the number of times the mouse stands on its hind feet, were recorded.

**Accelerating rotarod test**

Motor coordination and equilibrium were tested on an accelerating rotarod (Rotarod, Bioseb, Vitrolles, France). After training at constant speed (4 rpm, 2 min), three 5 min trials were performed with 5 min intertrial intervals. During these latter trials, mice were placed on a rotating rod (3 cm diameter) that accelerated from 4 to 40 rpm in 5 min, and the time until they fell from the rod was recorded.

**Supplementary References**

Johnston D, Miao-Sin Wu S (1995) Extracellular field recordings. In: Johnston D and Miao-Sin Wu S (Eds) Foundations of Cellular Neurophysiology, MIT Press, Cambridge Mass, pp 423-439

Kelai S, Renoir T, Chouchana L, Saurini F, Hanoun N, Hamon M, Lanfumey L (2008) Chronic voluntary ethanol intake hypersensitizes 5-HT(1A) autoreceptors in C57BL/6J mice. *J Neurochem* 107:1660-1670.

Mlinar B, Mascalchi S, Morini R, Giachi F, Corradetti R (2008) MDMA induces EPSP-Spike potentiation in rat ventral hippocampus in vitro via serotonin and noradrenaline release and coactivation of 5-HT4 and beta1 receptors. *Neuropsychopharmacology* 33:1464-1475.

Mlinar B, Stocca G, Corradetti R (2014) Endogenous serotonin facilitates hippocampal long-term potentiation at CA3/CA1 synapses. *J Neural Transm* (in press).

Tordoff MG, Bachmanov AA (2003) Influence of the number of alcohol and water bottles on murine alcohol intake. *Alcohol Clin Exp Res* 27:600-606.
