# Supplementary material for: Brain plasticity and cognitive functions after ethanol consumption in C57BL/6J mice
Source: Transl Psychiatry. 2015 Dec 15;5(12):e696–. doi: 10.1038/tp.2015.183 (PMC5068583; doi:10.1038/tp.2015.183)
Supplement: Supplementary Legends [file tp2015183x2.docx]

**Supplementary Legends**

**Figure S1**

**Ethanol consumption by C57BL/6J mice during the 21 day free-choice period**

Mice were offered 3% v/v ethanol (EtOH) versus water for 4 days, then 6% ethanol versus water for the next 4 days, and finally animals had access to 10% ethanol versus water for the subsequent 13 days (stabilization period). Positions of the tubes filled with ethanol or water were changed every two days to avoid possible preference bias. Food (standard mouse pellets A03; UAR, Strasbourg, France) was given ad libitum. Absolute grams of ethanol intake/kg body weight/24 h were determined for each mouse. Each point is the mean ± S.E.M. of 86 independent determinations. During the stabilization period, daily ethanol quantity consumed was 10.52 ± 0.30 g/kg/day (mean ± S.E.M., n = 86).

**Figure S2**

**Anxiety-like behavior in “ethanol” and “water” mice**

(A) Mice were subjected to the elevated plus-maze and the number of entries and time spent in the open arms were recorded over a 5 min period. Neither the number of entries and the time spent in the open arms was changed in “ethanol” compared to “water” mice (p = 0.196 and p = 0.977, respectively, Student’s t test). Each bar is the mean ± S.E.M. of n = 8 (“water”) and n = 10 (“ethanol”) mice.

(B) Mice were placed in an open field and the distance covered, the times spent at the periphery and at the centre were recorded over a 15-minute period. The distance covered and the time spent in the central area were not different in “ethanol” mice compared to “water” mice (p = 0.260, p = 0.731; respectively, Student’s t test). Each bar is the mean ± S.E.M. of n = 8 (“water”) and n = 10 (“ethanol”) mice.

**Figure S3**

**Primary latency to reach target hole in “ethanol” and “water” mice in the Barnes maze**

Mice were placed on the platform, the primary latency and the distance travelled to reach the escape box was measured for days of learning (A and B) and days of reversal learning (C and D).

(A and B) A two-way ANOVA revealed no effect of ethanol intake, a significant effect of time and no interaction between factors for the primary latency and the distance travelled to reach the target (Primary latency, F_(1,155)_ = 0.129, p = 0.720; F_(8,155)_ = 17.340, p < 0.0001; F_(8,155)_ = 0.696, p = 0.695; respectively; Distance travelled, F_(1,118)_ = 0.493, p = 0.484; F_(8,118)_ = 6.383, p < 0.0001; F_(8,118)_ = 1.271, p = 0.265; respectively). Each point is the mean ± S.E.M. of n = 10 (“water”) and n = 9 (“ethanol”) mice.

(C and D) A two-way ANOVA revealed no effect of ethanol intake, a significant effect of time and no interaction between factors for the primary latency and the distance travelled to reach the target (Primary latency, F_(1,57)_ = 0.137, p = 0.713; F_(3,57)_ = 3.020, p = 0.037; F_(3,57)_ = 0.339, p = 0.797; respectively; Distance travelled, F_(1,57)_ = 0.096, p = 0.757; F_(3,57)_ = 3.557, p = 0.019; F_(3,57)_ = 0.164, p = 0.920; respectively). Each point is the mean ± S.E.M. of n = 9 (“water”) and n = 9 (“ethanol”) mice.

**Figure S4**

**Locomotor activity and motor coordination in “ethanol” and “water” mice.**

(A) Mice were placed in actimetry boxes and the total locomotor activity and the number of rearing and beam crossing were measured during a 1-hour period. A two-way ANOVA analysis showed an effect of time on spontaneous locomotor activity in the actimeter, no effect of chronic ethanol intake and no interaction between variables (F_(11,143)_ = 48.950, p < 0.0001; F_(1,143)_ = 0.302, p = 0.592; F_(11,143)_ = 1.364, p = 0.196; respectively, repeated measures, two-way ANOVA). The numbers of rearing and beam crossing were not affected by chronic ethanol consumption (p = 0.961; p = 0.795, respectively, Student’s t test). Each point and bar are the mean ± S.E.M. of n = 6 (“water”) and n = 9 (“ethanol”) mice.

(B) Mice were placed on an accelerating rotarod, and the latency to fall was measured. Mice were tested for 3 consecutive trials of 5 min with an inter-trial interval of 5 min. A two-way ANOVA revealed a significant effect of trials on the latency to fall but no effect of ethanol consumption and no interaction between factors (F_(2,32)_ = 12.38, p = 0.0001; F_(1,32)_ = 0.462, p = 0.506; F_(2,32)_ = 0.174, p = 0.841; repeated measures, two-way ANOVA). Each bar is the mean ± S.E.M. of n = 8 (“water”) and n = 10 (“ethanol”) mice.
