# Supplementary material for: Brain plasticity and cognitive functions after ethanol consumption in C57BL/6J mice
Source: Transl Psychiatry. 2015 Dec 15;5(12):e696–. doi: 10.1038/tp.2015.183 (PMC5068583; doi:10.1038/tp.2015.183)
Supplement: Supplementary Tables [file tp2015183x3.docx]

| **Experiments** | **Ethanol consumption (g/kg/day) during stabilization period with 10% ethanol in drinking water** | **Total fluid consumed (g/kg/day)** | **Number of mice** |
| --- | --- | --- | --- |
| **Behavioral testing**  **(OF, Rotarod, FST)** | 10.92 ± 0.63 | 182.10 ± 18.24 | Water n=8  Ethanol n=10 |
| **Behavioral testing (actimeter)** | 10.74 ± 0.35 | 170.20 ± 15.66 | Water n=6  Ethanol n=9 |
| **Behavioral testing**  **(Novel object recognition)** | 10.60 ± 0.53 | 135.30 ± 10.25 | Water n=6  Ethanol n=9 |
| **Behavioral testing**  **(Fear conditioning)** | 10.46 ± 0.36 | 142.70 ± 13.12 | Water n=6  Ethanol n=8 |
| **Behavioral testing**  **(Barnes Maze)** | 10.93 ± 0.51 | 137.00 ± 13.23 | Water n=10  Ethanol n=10 |
| **RT-qPCR** | 10.04 ± 0.39 | 140.00 ± 17.84 | Water n=6  Ethanol n=9 |
| **Immunoblotting** | Vehicle-treated 9.54 ± 0.88 | 139.50 ± 17.70 | Water n=6  Ethanol n=9 |
|  | ANA-12-treated 8.87 ± 0.77 | 134.00 ± 13.53 | Water n=6  Ethanol n=9 |
| **MeDIP** | 11.60 ± 0.24 | 153.90 ± 14.51 | Water n=6  Ethanol n=9 |
| **LTP recordings** | 10.17 ± 0.17 | 133.60 ± 2.19 | Water n=15  Ethanol n=13 |

**Table S1:** Ethanol intake and total fluid consumed within the different groups of mice having free access to 10% ethanol that had been included in the experimental series. A one-way ANOVA did not reveal differences between groups in both ethanol consumption and in total fluid consumed (Ethanol, p = 0.172; Total fluid, p = 0.060).

| **mRNA expression analysis** | | |
| --- | --- | --- |
| **Genes** | **5’ primer** | **3’ primer** |
| *Dnmt1* | AGCCGCTCAAAGCAAAAGTG | TGGGGTTCATCCACAGCATC |
| *Dnmt3a* | TCAATGTCACCCTGGAGCAC | CTGCAGCAGTTGTTGTTCCC |
| *Dnmt3b* | GATGAGGAGAGCCGAGAACG | CAGAGCCCACCCTCAAAGAG |
| *Hprt* | GATGATGAACCAGGTTATGAC | GTCCTTTTCACCAGCAAGCTTG |
| *β-actin* | CCACCATGTACCCAGGCATT | CGGACTCATCGTACTCCTGC |

**Table S2**: Primer sequences used for quantitative real-time PCR analysis of mRNAs with Absolute SYBR Green technology.

| **Methylated DNA immunoprecipitation (MeDIP) analysis** | | |
| --- | --- | --- |
| ***Bdnf* CpG islands** | **5’ primer** | **3’ primer** |
| *I* | CGCAGTCACTAGTGGGAAGTGTA | GTGTAGGCTAGGAGGGAAGAT |
| *II* | AACGTAAGGAAGTGGAAGAAACC | ATGAAGTACTACCACCTCGGACA |
| *III* | GGAAGGTCCTTTCTAGATCGAAG | TACCAGGCCACCCAGGTAGT |
| *IV* | CGTTTTCTCAGTCAGATCTACGC | TCCGTATTTAAAACAGCACCATC |
| *V* | CTATTTCGAGGCAGAGGAGGTAT | GAGAAGCTCCATTTGATCTAAGGC |
| *VI* | CTAGGGGACTGAGAAGTTGTGG | TTTCTCTCACACTGAAGGGATTC |
| *VII* | TTGGAGCCTCCTCTACTCTTTCT | AGACATGTCCACTGCAGTCTTTT |
| *Gapdh* | CTCCCAGGAAGACCCTGCTT | GGAACAGGGAGGAGCAGAGA |

**Table S3**: Primer sequences used for MeDIP analysis. The CpG island was defined as follow: island size > 200 bp, GC Percent > 50.0%, observed/expected > 0.6.
