# Supplementary figures and images for: Brain plasticity and cognitive functions after ethanol consumption in C57BL/6J mice
Source: Transl Psychiatry. 2015 Dec 15;5(12):e696–. doi: 10.1038/tp.2015.183 (PMC5068583; doi:10.1038/tp.2015.183)

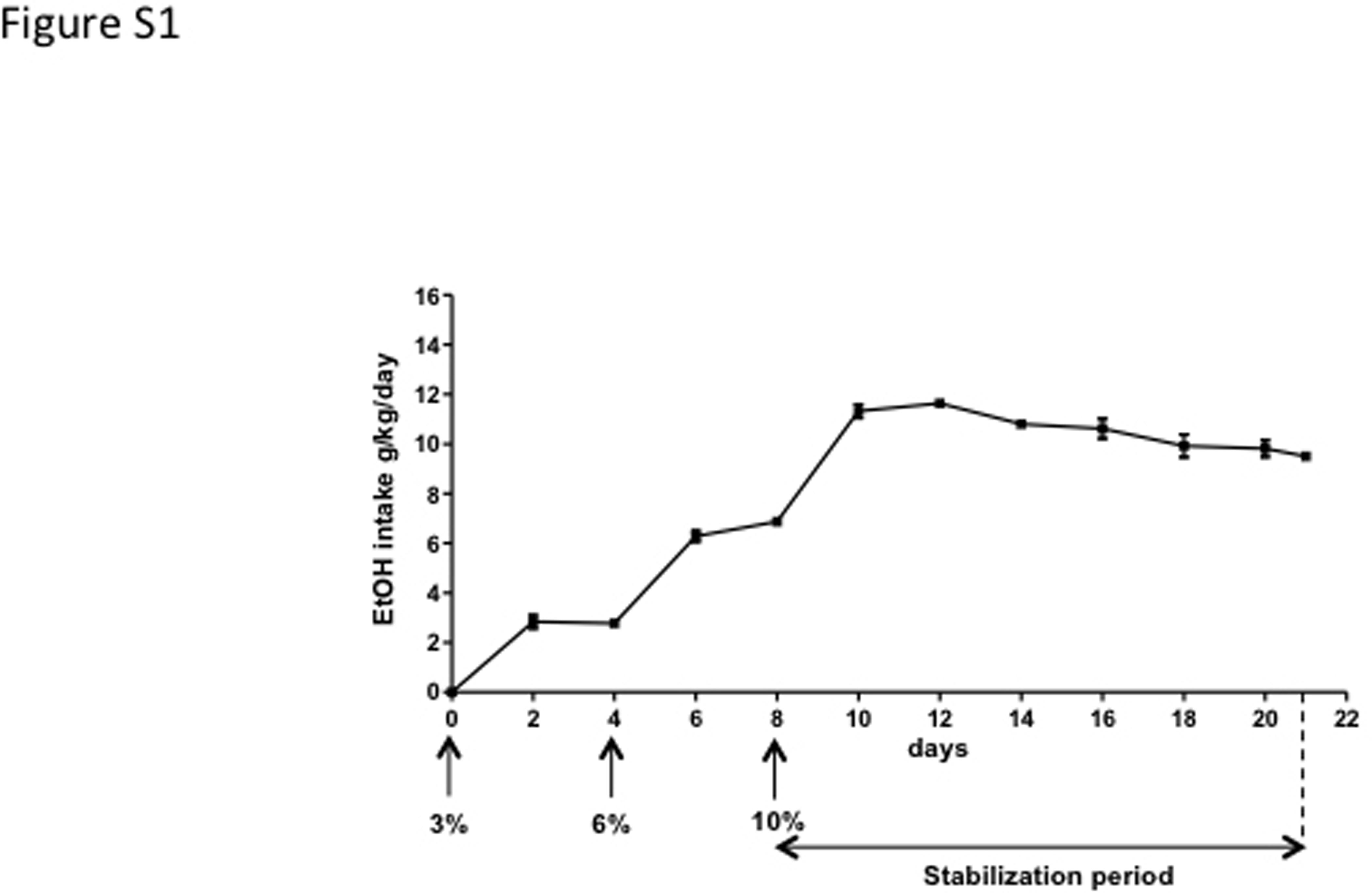

Supplement: Supplementary Figure S1 [file tp2015183x4.tif]

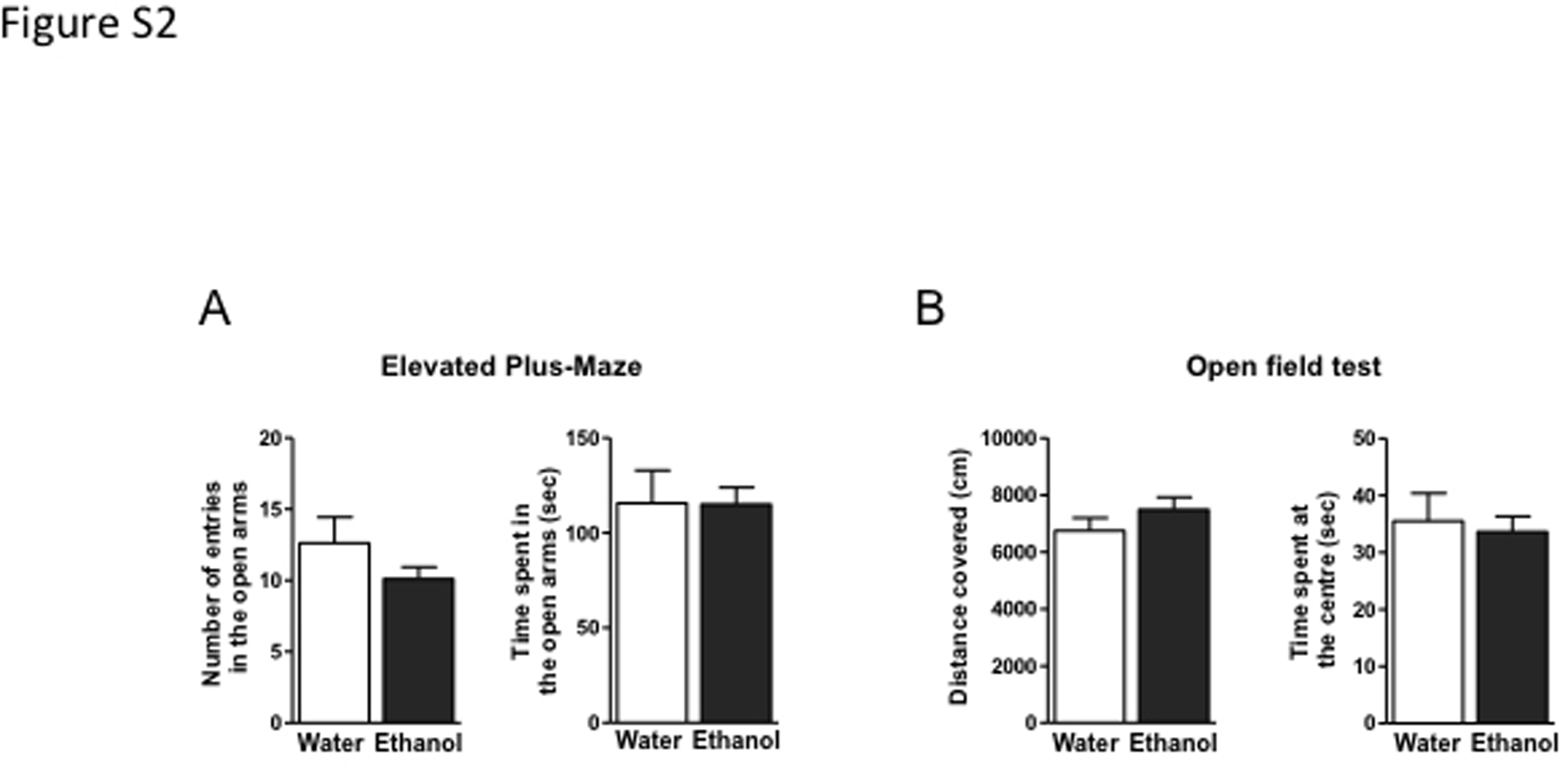

Supplement: Supplementary Figure S2 [file tp2015183x5.tif]

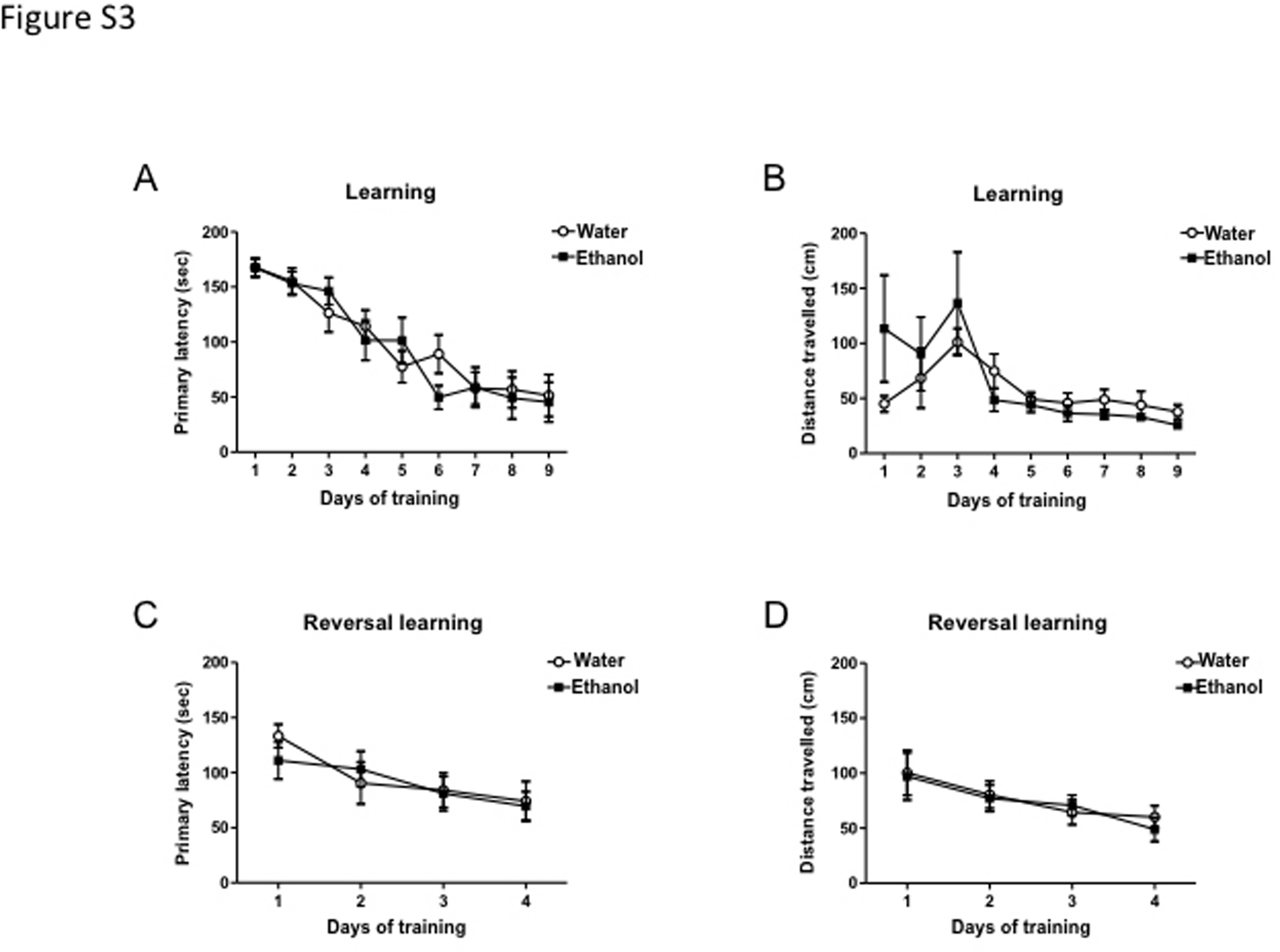

Supplement: Supplementary Figure S3 [file tp2015183x6.tif]

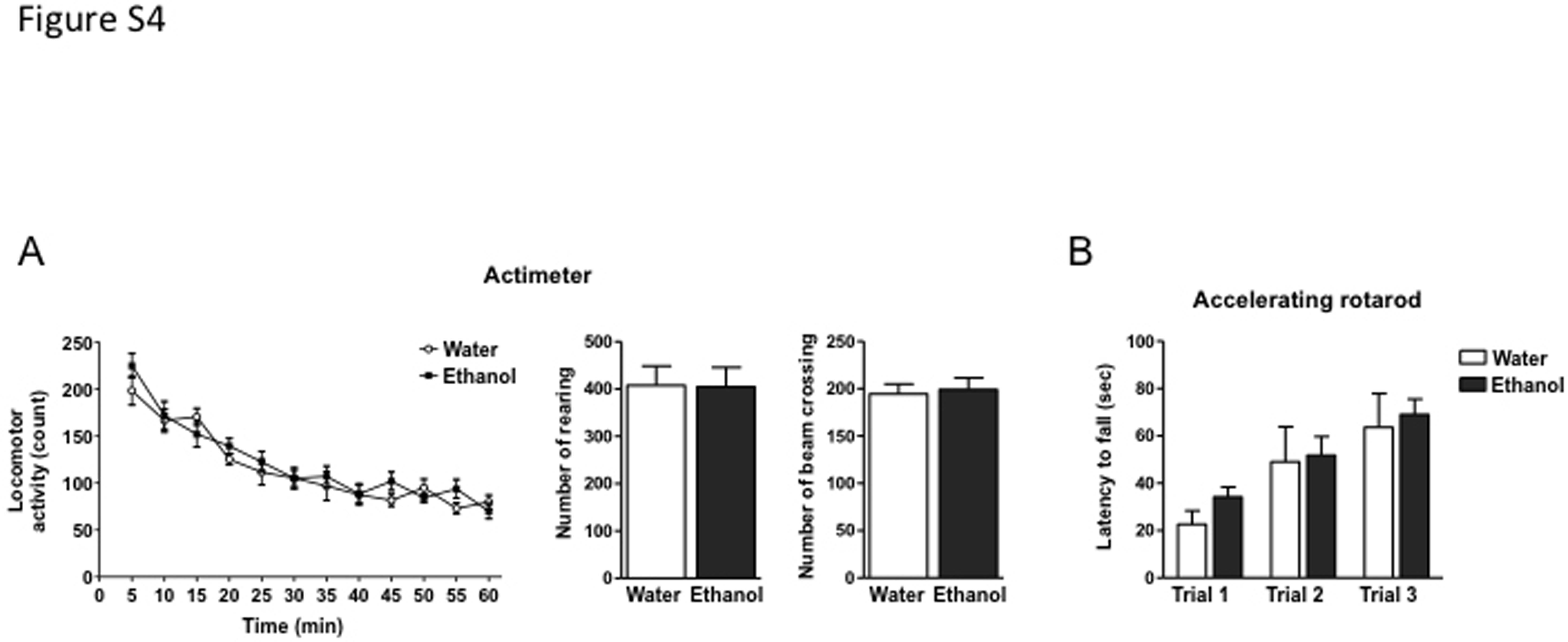

Supplement: Supplementary Figure S4 [file tp2015183x7.tif]
